# Keystone Veterinary Medical Association

**Published:** 1895-01

**Authors:** W. L. Rhoads

**Affiliations:** Secretary


Secretary.

### KEYSTONE VETERINARY MEDICAL ASSOCIATION.

The regular monthly meeting of the Keystone Veterinary Medical Association was held on Tuesday evening, January 8, 1895, at the office of Dr. Jas. B. Rayner, West Chester, Pa.

President Lintz called the meeting to order, and after roll-call by Secretary Rhoads the report of the Legislative Committee was called for. The Chairman, Dr. Hoskins, before summarizing the progress made in advancing the legislation looking to the establishment of a State Live Stock Sanitary Commission, read from the Philadelphia *Times* of the 7th inst. a special telegram from Harrisburg, stating that this bill had been so drawn as to make ineligible to appointment Dr. Francis Bridge, who is now employed by the State Board of Agriculture. This statement was characterized by Chairman Hoskins as a wilful, deliberate, malicious lie, and that those who furnished the information to the *Times* correspondent knew it to be so. He referred to such methods of warfare as being evidence of a weak cause, and that they would react against those who resorted to their use in bolstering up a weak movement. The most encouraging reports were made from a score of counties and districts. Drs. Rayner, Rhoads, and McAnulty all spoke encouragingly of the favorable responses they had met with and of the general approval they received in endeavoring to place these questions under the control and direction of a body of experts.

The subject for the half-hour talk was then opened, and Dr. Hoskins being accorded the floor, cited two cases of cerebro-spinal meningitis having come under his notice during the month, one of these proving fatal in a sorrel gelding used as a road horse. An interesting fact in conjunction with this case was the rapid loss of weight. On admission to his sanitarium the scales recorded a weight of 930 pounds; for eighteen hours the weight remained stationary; twenty-four hours later he weighed but 888 pounds; the next twenty-four hours recorded a further loss of 24 pounds, making his weight 864 pounds; eight hours later the horse assumed the recumbent position and paralysis became almost complete; estimated loss of weight 50 pounds, when it was deemed wise to destroy him.

The second case showing only incipient symptoms of lassitude, slight dragging of toes of hind limbs, very dry condition of feces; stationary in weight for the first fifteen hours, gaining successively four, six, ten, ten, and five pounds the following five days. In the first case inability to swallow water became complete after twenty-four hours. The second case was able at all times to drink. Two mules had died in the stable where these cases developed, some three weeks before from the same disease.

The probable causes as to food, drainage, improper light, ventilation, were discussed by Drs. Rayner, Lintz, and McAnulty, and its propagation through the atmosphere as a media was referred to specially by Dr. Lintz.

The lateness of the hour prevented Dr. Jas. B. Rayner from presenting his paper, and it was postponed to the February meeting.

W. L. RHOADS,
Secretary.

